# Biodiversity of poly-extremophilic *Bacteria*: Does combining the extremes of high salt, alkaline pH and elevated temperature approach a physico-chemical boundary for life?

**DOI:** 10.1186/1746-1448-5-9

**Published:** 2009-11-23

**Authors:** Karen J Bowers, Noha M Mesbah, Juergen Wiegel

**Affiliations:** 1 Department of Microbiology, University of Georgia, Athens, GA, USA

## Abstract

Bacterial microorganisms that grow optimally at Na^+ ^concentrations of 1.7 M, or the equivalent of 10% (w/v) NaCl, and greater are considered to be extreme halophiles. This review focuses on the correlation between the extent of alkaline pH and elevated temperature optima and the extent of salt tolerance of extremely halophilic eubacteria; the focus is on those with alkaline pH optima, above 8.5, and elevated temperature optima, above 50°C. If all three conditions are required for optimal growth, these microorganisms are termed "poly-extremophiles". However, only a very few extreme halophiles able to grow optimally under alkaline conditions as well as at elevated temperatures have been isolated so far. Therefore the question is: do the combined extreme growth conditions of the recently isolated poly-extremophiles, *i.e*., anaerobic halophilic alkalithermophiles, approach a physico-chemical boundary for life? These poly-extremophiles are of interest, as their adaptive mechanisms give insight into organisms' abilities to survive in environments which were previously considered prohibitive to life, as well as to possible properties of early evolutionary and extraterrestrial life forms.

## Extremely halophilic *Bacteria*

It is frequently asked: what are the physical and chemical boundaries for life? How extreme can conditions become and still support life? In respect to one extreme--haline conditions--the answer is simple; growth has been observed at saturated concentrations of sodium salts, mainly NaCl. But what happens if one tries to increase the solubility of these salts by increasing the temperature? This overview deals with the diversity of bacteria able to grow under concomitant extreme growth conditions, namely high sodium salt concentration, alkaline pH and elevated temperature, and thus with the recently isolated anaerobic halophilic poly-extremophiles.

Different authors use different definitions for what constitutes a halophile; one definition identifies microorganisms which grow optimally at Na^+ ^concentrations greater than 0.2 M as halophiles [1]. For this review, the authors wish to focus upon bacterial microorganisms that grow at the upper limits of the combined extremes, and in the case of halophiles, optimally at Na^+ ^concentrations of 1.7 M, or the equivalent of 10% NaCl. These microorganisms are defined as extreme halophiles, in contrast to those microorganisms which merely tolerate such sodium concentrations or which grow optimally at marine salt concentrations of approximately 3.5% w/v (Table 1). In this review Na^+ ^concentrations are given in mol/liter, rather than % NaCl, since some of the alkaliphilic halophiles require media with carbonates, usually provided as sodium carbonates, for pH control [2,3]. Therefore the [Na^+^] comes both from the sodium carbonates and from the supplemented NaCl. Many of the well-known extreme halophiles are *Archaea *(over one hundred species in the family *Halobacteriacae *alone); however, some extremely halophilic bacteria have been described [see Additional File 1], and this review will focus exclusively on extremely halophilic *Bacteria*. These bacteria have been isolated from various extreme environments such as solar thalassohaline salterns (*i.e*., originating from marine waters) [4], athalassohaline (*e.g*., Wadi An Natrun, Egypt) [2] and ancient thalassohaline (*e.g*., Great Salt Lake, UT, USA) [5] salt lakes, marine environments [6], and fermented fish sauces [7]. Additionally, among the validly published taxa (*i.e*., published in, or publication validated by, the *International Journal of Systematic and Evolutionary Microbiology*), the extremely halophilic *Bacteria *are relatively equally distributed between aerobic and anaerobic species, with the addition of four facultative anaerobes, such as *Halomonas sinaiensis *[8] and *Thiohalorhabdus denitrificans *[9]. Examples of extremely halophilic bacteria--representing different types of extrema--include *Halorhodospira halochloris *(basonym *Ectothiorhodospira*) [10], which, at 4.62 M, has one of the highest [Na^+^] optima [11]; *Halomonas taeanensis*, which is capable of growing over the unusually wide range of 0-5.13 M Na^+ ^[12]; and *Natranaerobius 'grantii'*, which tolerates saturated NaCl concentrations in its growth medium at elevated temperature and alkaline pH [13].

**Table 1:** Growth Characteristics of Extremophiles

Growth Characteristic	Minimum	Optimum	Maximum
Halotolerant	-	[Na^+^] < 0.2 M	[Na^+^] > 0.2 M
Halophilic	[Na^+^] ≥ 0.2 M	0.2 M < [Na^+^] < 1.7 M	-
Extreme	[Na^+^] ≥ 0.2 M	[Na^+^] ≥ 1.7 M	-
Alkalitolerant	pH ≥ 6.0	pH < 8.5	pH > 9.0
Alkaliphilic			
Facultative	pH < 7.5	pH ≥ 8.5	-
Obligate	pH ≥ 7.5	pH ≥ 8.5	pH ≥ 10.0
Thermotolerant	-	T < 50°C	T > 50°C
Thermophilic	-	T ≥ 50°C	T > 55°C

^a^pH should be measured at optimum growth temperature [25]

^b^10% added NaCl equals 1.7 M Na^+^

^c ^- indicates no boundary at this parameter

Bacterial extreme halophiles exhibit various physiological and nutritional properties [3,14-16] and belong to different phylogenetic groups such as the order *Actinomycetales *from the phylum *Actinobacteria*; the order *Sphingobacteriales *from the phylum *Bacteroidetes*; the orders *Bacillales*, *Halanaerobiales *and *Natranaeriobiales *from the phylum *Firmicutes*; the orders *Rhizobiales *and *Rhodospirillales *from the subphylum *α-Proteobacteria*; and the orders *Chromatiales*, *Oceanospirillales *and *Pseudomonadales *from the subphylum *γ-Proteobacteria*. Although many extreme halophiles are mesophilic or neutrophilic, moderately thermophilic extreme halophiles have been described, along with several alkaliphilic extreme halophiles. Microorganisms which have two extreme growth optima are generally described using the two specific extrema, *e.g*., alkaliphilic halophiles. However, only a very few extreme halophiles able to grow optimally under alkaline conditions as well as at elevated temperatures have been isolated so far. This review will focus on the pH and temperature optima of extremely halophilic bacteria, with a focus on those with alkaline pH optima, above 8.5, and elevated temperature optima, above 50°C. These microorganisms are considered extremophiles, and, if all three conditions are required for optimal growth, are termed by the authors "poly-extremophiles". They are of great interest, as their adaptive mechanisms give insight into the abilities of bacteria to survive in environments which were previously considered prohibitive to life, as well as to possible properties of early evolutionary and extraterrestrial life forms [17]. The purpose of this overview is to discover whether a correlation exists amongst the validly published bacterial taxa between the extents of halophily, alkaliphily and/or thermophily. In other words: is the extent of one extreme condition limiting the concomitant extent of other extreme growth condition (*e.g*., a higher temperature optimum requiring a less alkaline pH or a lower sodium salt concentration)?

Currently (September 2009), there are over sixty validly published species (*i.e*., published or validated in the *International Journal of Systematic Bacteriology/Systematic and Evolutionary Microbiology*, as listed at http://www.bacterio.cict.fr) which are extremely halophilic, according to the description. Of these species, approximately thirty percent have [Na^+^] optima of less than 2.0 M (equivalent to approximately 12% w/v NaCl), nineteen of which are published at 1.7 M (equivalent to approximately 10% w/v NaCl). Approximately forty-five percent of the extremely halophilic species have published [Na^+^] optima equal to or greater than 2.0 M but less than 3.4 M, and only thirteen microorganisms (approximately 25%) have published [Na^+^] optima equal to or greater than 3.4 M (equivalent to approximately 20% (w/v) NaCl). Additionally, approximately thirty percent of the species tolerate [Na^+^] 5.0 M or greater (equivalent to approximately 29% w/v NaCl). Among these microorganisms, only three--*Halorhodospira halochloris*, *Halanaerobium lacusrosei *and the unpublished *Natranaerobius 'grantii'*--have been described which grow in the presence of saturated NaCl (*i.e*., 5.5 to 6.5 M, since the saturation point is dependent upon media composition, growth pH and temperature) [11,13,18]. Clearly, as the [Na^+^] increases the number of known microorganisms with the adaptive mechanisms that enable them to thrive under these conditions decreases. However, while the number of microorganisms with [Na^+^] optima above 3.0 M is small, a significantly larger number of bacterial halophiles are able to tolerate 3.0 M [Na^+^]; in fact, all of the extreme halophiles with a published [Na^+^] maximum are able to do so [see Additional File 1].

## pH optima and ranges of extreme halophiles

**Figure 1:**
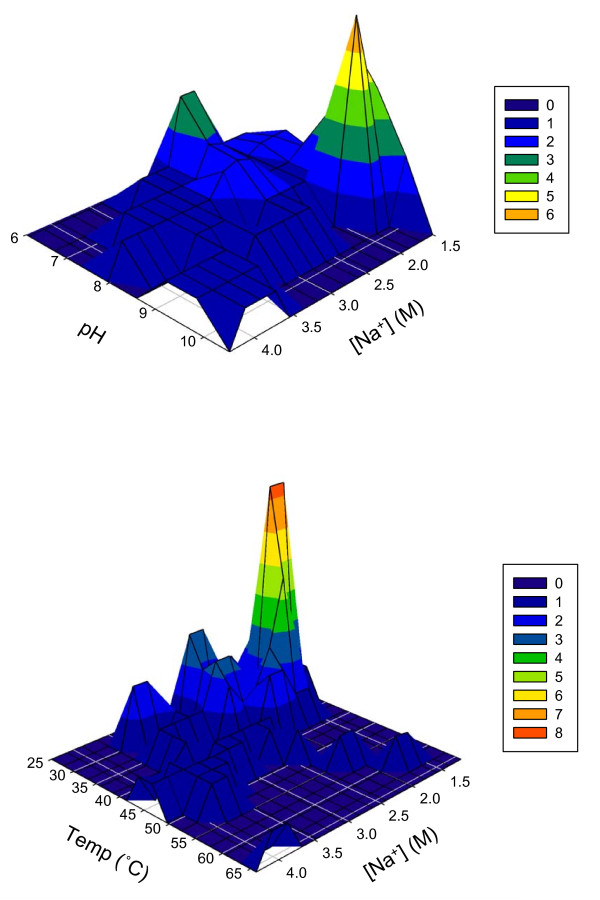
Correlation of [Na^+^] optimum and pH or temperature optima of extreme halophiles. Figure 1a. **Correlation between [Na^+^] optimum and pH optimum.** [Na^+^] optimum (M) is plotted against pH optimum; number of microorganisms at each locus is plotted on z-axis as indicated by color coding; no representation (zero) is indicated in the darkest shade. Figure 1b. **Correlation between [Na^+^] vs. temperature optima.** [Na^+^] optimum (M) is plotted against temperature optimum (°C); number of microorganisms at each locus is plotted on z-axis as indicated by color coding; no representation (zero) is indicated in the darkest shade.

Interestingly, out of all the established extremely halophilic bacteria, only nineteen species have pH optima of 8.5 or greater [see Additional File 1], although many salt lakes and salterns from which these organisms were isolated have alkaline pH values. Of these, only ten species combine an elevated pH optimum with a [Na^+^] optimum of 2.0 M or greater. The distribution of the [Na^+^] and pH optima are shown in Figure 1a; clearly, the combinations of pH optimum 9 with a [Na^+^] optimum of approximately 1.7 M is the most highly represented, followed by the combinations of pH optima 7 and 8 with [Na^+^] optimum of approximately 1.7 M. Theoretically, if the adaptive resources of a microorganism are being utilized heavily to deal with one type of environmental stress (*e.g*., osmotic stress) there will be less available resources to deal with other types of environmental stressors: in this case, the elevated pH. Microorganisms with pH values for optimal growth above 8.5 carry with them the usual energetic problems of alkaliphiles, *e.g*., an inverted pH gradient and thus a suboptimal proton motive force [3]. In the case of extremely halophilic alkaliphiles these problems are exacerbated by the need to keep the intracellular sodium concentration below toxic levels, which is frequently as low as a few mM [19,20]. This complication may explain the more prevalent occurrence of microorganisms growing optimally in environments that are pH neutral or near neutral. However, it could also be an artifact of the fact that researchers have investigated the alkaline halobiotic environments and the biodiversity of their microorganisms less than those of neutral or slightly acidic halobiotic environments. Mesbah *et al *[21] have shown that the biodiversity of the alkaline athalassohaline lakes of Wadi An Natrun (North Egypt) is relatively high. Similar observations were made by Ghozlan *et. al*. regarding saline habitats of Alexandria, Egypt [22] and Duckworth *et. al*. regarding various alkaline soda lakes [23]. Obligately alkaliphilic halophilic bacteria from the lakes of the Wadi An Natrun include: *Natranaerobius thermophilus *[2], *Natranaerobius trueperi *and *Natronovirga wadinatrunensis *[24]. Each of these microorganisms has a pH^55°C ^optimum of 9.5 or greater as well as a [Na^+^] optimum greater than 2.5 M. As previously recommended by Wiegel [25], the pH values for *N. thermophilus*, *N. 'jonesii'*, *N. trueperi*, *N. wadinatrunensis *and *N. 'grantii' *were measured at each microorganism's optimum growth temperature, denoted with a superscript (*i.e*., pH^55°C^). The pH measurement of an alkaline, complex growth medium which is at an elevated temperature (*i.e*., 55°C) with a pH probe calibrated at a much lower temperature (*i.e*., 25°C) will yield a pH measurement that can be upwards of one unit greater than that measured with a pH probe calibrated at the elevated temperature [25]. A number of other species have pH optima of 8.5-9 or greater, and of these, three species--*Halorhodospira halochloris *[10,11], *Halorhodospira abdelmalekii *[11,26] and *Natroniella acetigena *[27]--also have [Na^+^] optima of greater than 2.5 M, whereas the more-studied *Salinibacter ruber*, with a [Na^+^] optimum around 4 M, grows optimally at pH 8.0 and does not grow above pH 8.5 [28]. Overall, the number of anaerobic and aerobic haloalkaliphiles is similar (there are nine anaerobic and eleven aerobic haloalkaliphiles); interestingly, however, of the group of organisms just discussed, which have a [Na^+^] optimum greater than 2.5 M as well as a pH optima greater than 8.5, all are obligately anaerobic with a fermentative metabolism.

## Elevated temperature optima and ranges of extreme halophiles

The other environmental stressor of interest is elevated temperature. Figure 1b contrasts, in similar fashion to Figure 1a, the correlation between temperature optima with the [Na^+^] optima of the halophiles under review. Elevated temperature optima--which is, for true thermophiles, above 50°C--are even more infrequent amongst the published extremely halophilic eubacteria than are alkaline pH optima. An additional problem for thermophiles is that, at the elevated temperature, the cell membrane becomes more permeable to the diffusion of protons and to increased Na^+ ^diffusion [29]--especially in saline environments--making it more difficult for the cell to keep the intracellular [Na^+^] at a millimolar level against the molar extracellular concentration of Na^+^. The increased Na^+ ^permeability is less pronounced than the increased proton permeability, but is significant in extremely halophilic conditions. Of the extremely halophilic bacteria with determined temperature optima, only eight have temperature optima equal to or greater than 50°C. *Dichotomicrobium thermohalophilum *and *Halorhodospira halophila *have published temperature optima of 50°C [30,31], *Halonatronum saccharophilum *of 55°C [32], *Halothermothrix orenii *of 60°C [33], the unpublished *Natranaerobius 'jonesii' *of 66°C [13], *Natranaerobius thermophilus *of 53°C [2], *Natranaerobius trueperi *of 52°C [24], and *Natronivirga wadinatrunensis *of 51°C [24]. Three others, *Salinibacter ruber*, *Halorhodospira halochloris *and the unpublished *Natranaerobius 'grantii'*, are thermotolerant, and have temperature optima of 46-48°C, just below the thermophilic designation [see Additional File 1]. Of these two groups of organisms only *D. thermohalophilum *and *S. ruber *are aerobes; the remaining organisms are all obligately anaerobic. While there are few true thermophilic extreme halophiles, many species--approximately forty percent of the validly published halophiles--are able to tolerate temperatures above 50°C [see Additional File 1]. Most species (60%) have temperature optima of 40°C or below: twenty-five percent of these have temperature optima of 38-40°C, and approximately fifty percent have temperature optima of 32-37°C. The remaining twenty-five percent have temperature optima between 30 and 32°C. It is important to note that not all species considered have published temperature optima. Interestingly, no strictly psychrophilic (T_opt _≤ 15°C and T_max _≤ 20°C) extreme halophiles have been described to date, though many psychrophiles and psychrotolerant species, such as *Psychrobacter okhotskensis *(T_range _5-35°C, T_opt _25°C), tolerate up to 4 M Na^+ ^[34]; however the published [Na^+^] optima of these microorganisms are below 1.7 M.

## Poly-extremophiles: extreme halophiles with elevated temperature optima and alkaline pH optima

**Figure 2:**
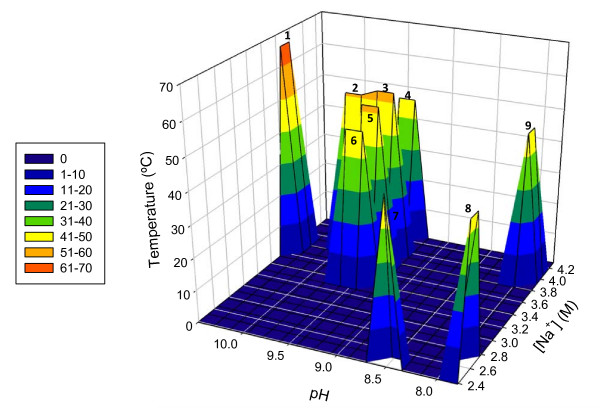
**Clustering of poly-extremophiles relative to other extreme halophiles**. Representation of extremely halophilic *Bacteria *for which both additionally-considered growth conditions (pH and temperature) approach or exceed thermophilic and alkaliphilic levels. The optima of the discussed recently isolated poly-extremophiles cluster in the upper range for each criterion, well-separated from other representative microorganisms. The z-axis color coding depicts the temperature optimum of each bacterium. Species represented: 1. *Natranaerobius 'jonesii'*, 2. *Natronovirga wadinatrunensis*, 3. *Natranaerobius thermophilus*, 4. *Natranaerobius 'grantii'*, 5. *Natranaerobius truperi*, 6. *Halorhodospira halochloris*, 7. *Dichotomicrobium thermohalophilum*, 8. *Halanaerobacter salinarius*, 9. *Salinibacter ruber*

Of the group of thermophilic extremely halophilic *Bacteria*, only the anaerobic microorganisms *Natranaerobius thermophilus*, *Natranaerobius 'jonesii', Natranaerobius trueperi *and *Natronovirga wadinatrunensis *demonstrate elevated pH optima (9.5-10.5) and [Na^+^] optima (3.7-3.9 M), a group we term poly-extremophiles. Also of note are *Halorhodospira halochloris *which has a slightly lower temperature optimum of 48°C, a pH optimum of 8.5 and a [Na^+^] optimum of 4.62 M and *Natranaerobius 'grantii' *which has a temperature optimum of 46°C, a pH^45°C ^optimum of 9.5 and [Na^+^] optimum of 4.3 M [see Additional File 1]. The uniqueness of these poly-extremophiles when compared to other known, extremely halophilic *Bacteria *is illustrated in Figure 2. On all three axes, [Na^+^], pH and temperature, these bacteria fall much farther along the axis than do other extreme halophiles. The fact that these microorganisms can not only survive but thrive under these multiple extreme conditions has extended the known boundaries for life at a combination of multiple extrema. Microorganisms living in extreme environments utilize a number of adaptive mechanisms in order to enable them to proliferate, and this is true to an even greater extent of poly-extremophiles. Cytoplasmic acidification for pH adaptation under halophilic growth conditions using multiple monovalent cation/proton antiporters with various pH ranges [35], the combined use of organic compatible solutes [36] and intracellular accumulation of K^+ ^[20] for adaptation to osmotic pressure are three of the adaptive mechanisms employed by this group of microorganisms [1,3,37].

To date, the only true anaerobic poly-extremophiles have been isolated from sediment samples taken from one of two locations. Three of the four anaerobic extremely halophilic alkalithermophiles, *N. thermophilus*, *N trueperi *and *N. wadinatrunensis*, were isolated from the solar-heated, alkaline, hypersaline lakes of the Wadi An Natrun, Egypt (temperatures up to 60°C measured in the salt brine) [2,24]. The Wadi An Natrun is a series of eight lakes in northern Egypt noted for their salinity and alkaline pH. *Halorhodospira halochloris*, an anaerobic phototrophic purple bacterium, which is a thermotolerant, rather than thermophilic alkaliphilic halophile, was also isolated from the Wadi An Natrun [11]. *N. 'jonesii'*, and the thermotolerant *N. 'grantii'*, were isolated from sediment samples from Lake Magadi, in the Kenyan Rift Valley [13]. Lake Magadi, like the lakes of the Wadi An Natrun, is noted for its salinity and alkalinity. In places, the temperature of the lake exceeds 45°C; however, the lake is fed by saline hot springs in addition to being heated by sun rays.

The question remains: do the physiological and bioenergetic demands of dealing with one extreme condition (*e.g*., elevated temperature) limit an organisms' ability to meet the demands of two additional extreme conditions (*e.g*., elevated pH and high sodium concentrations) or reduce the extent of the other extrema to lower levels? A first look at the distribution of the peaks in Figure 2 suggests that yes, amongst the presented microorganisms, the more extreme the optimum is for one condition, the less extreme the optima for other conditions tend to be. However, the recent isolation and cultivation of the anaerobic poly-extremophiles in the novel order *Natranaerobiales*, which grow with doubling times around 3-4 h [2,13,24] and which require elevated temperatures, alkaline pH and high [Na^+^] in order to survive, demonstrate that microorganisms can thrive under combinations of multiple extrema. Furthermore, there is a notion that aerobic extremophiles, with higher ATP yields via electron transport phosphorylation should be better suited to extreme conditions than are anaerobic fermentative extremophiles, which have significantly lower ATP yields per substrate utilized, but so far there are no validly published aerobic *Bacteria *with similar combinations of growth conditions such as those of the anaerobic *Natranaerobius *species. The point needs to be stressed that with further investigation into alkaline halobiotic environments more, possibly many more, bacterial poly-extremophiles will be isolated and identified. As noted by Foti *et al*. (2008), few species identified from hypersaline habitats via culture independent methods are closely related to validly described species. Additionally, in the study performed by Foti *et al*. (2008) on Russian soda lakes, none of the validly described species were defined as haloalkaliphilic or haloalkalitolerant [38]; however, other studies on Kenyan and Egyptian soda lakes have identified uncultured clones related to validly described haloalkaliphiles [21,39]. While the utility of culture independent methods cannot be disputed, the presence of a microorganism in an environment does not necessarily imply that the particular environment under investigation represents the optimal environment for the growth of the microorganism. The only way to learn the true ranges and optimum growth conditions for a particular species is to characterize the cultured species, therefore limiting our discussion to validly published microorganisms. Although nearly every month novel halophiles are published in the *International Journal of Systematic and Evolutionary Microbiology *the majority of them are *Archaea*, or grow only at slightly elevated salt concentration, and are not bacterial extreme halophiles. The authors predict that when investigators focus more on isolating extreme halophiles, and especially poly-extremophilic halophiles, from extreme habitats the present list will be significantly extended, and the present limits of combined alkaline pH, elevated temperature and [Na^+^] could be pushed to more extreme values. Then the questions arise: what are the final boundaries? Does a super bacterium exist which can grow at the presently known limit of alkalinity, around pH 12 (or conversely, at acidity of around pH 1); at the present limit of temperature, around 121°C (or conversely, at temperatures below -12°C); as well as at saturated [Na^+^]? Thus far, no single aerobic or anaerobic bacterial or archaeal isolate has been found with optima even near these levels; however, this lack of knowledge certainly does not rule out the existence of such a microorganism. The authors hope that this overview will stimulate further investigation and isolations of these intriguing poly-extremophilic bacterial halophiles and elucidation of their unique physiological and biochemical properties for biotechnological applications.

## Competing interests

The authors declare that they have no competing interests.

## Authors' contributions

All authors drafted and reviewed the initial manuscript. All authors read and approved the final manuscript.

## Supplementary Material

Additional file 1 **[Na^+^], pH and Temperature Optima and Ranges for Bacterial Extreme Halophiles**. The data provided show the [Na^+^], pH and temperature optima and ranges for validly published bacterial extreme halophiles. Click here for file

## References

[B1] Oren A Dworkin M, Falkow S, Rosenberg E, Schleifer K-H, Stackebrandt E Life at high salt conditions The Prokaryotes. A Handbook on the Biology of Bacteria: Ecophysiology and Biochemistry 2006 2 New York: Springer 263 282

[B2] Mesbah NM Hedrick DB Peacock AD Rohde M Wiegel J *Natranaerobius thermophilus *gen. nov., sp. nov., a halophilic, alkalithermophilic bacterium from soda lakes of the Wadi An Natrun, Egypt, and proposal of *Natranaerobiaceae *fam. nov. and *Natranaerobiales *ord. nov Int J Syst Evol Microbiol 2007 57 2507 2512 10.1099/ijs.0.65068-0 17978210

[B3] Mesbah NM Wiegel J Wiegel J, Maier RJ, Adams MWW Life at extreme limits: the anaerobic halophilic alkalithermophiles Incredible Anaerobes: from Physiology to Genomics to Fuels 2008 Boston: Blackwell Pub. on behalf of the New York Academy of Sciences 44 57 10.1196/annals.1419.028 18378586

[B4] Mouné S Manac'h N Hirschler A Caumette P Willison JC Matheron R *Haloanaerobacter salinarius *sp. nov., a novel halophilic fermentative bacterium that reduces glycine-betaine to trimethylamine with hydrogen or serine as electron donors; emendation of the genus *Haloanaerobacter* Int J Syst Evol Microbiol 1999 49 103 112 10.1099/00207713-49-1-103 10028251

[B5] Fendrich C *Halovibrio variabilis *gen. nov. sp. nov., *Pseudomonas halophila *sp. nov. and a new halophilic aerobic coccoid *Eubacterium *from Great Salt Lake, Utah, USA Syst Appl Microbiol 1988 11 36 43

[B6] Kim KK Jin L Yang HC Lee S *Halomonas gomseomensis *sp. nov., *Halomonas janggokensis *sp. nov., *Halomonas salaria *sp. nov. and *Halomonas denitrificans *sp. nov., moderately halophilic bacteria isolated from saline water Int J Syst Evol Microbiol 2007 675 681 10.1099/ijs.0.64767-0 17392185

[B7] Pakdeeto A Tanasupawat S Thawai C Moonmangmee S Kudo T Itoh T *Salinicoccus siamensis *sp. nov., isolated from fermented shrimp paste in Thailand Int J Syst Ev Microbiol 2007 57 2004 2008 10.1099/ijs.0.64876-0 17766863

[B8] Romano I Lama L Orlando P Nicolaus B Giordamo A *Halomonas sinaiensis *sp. nov., a novel halophilic bacterium isolated from a salt lake inside Ras Muhammad Park, Egypt Extremophiles 2007 11 789 786 10.1007/s00792-007-0100-3 17618404

[B9] Sorokin DY Tourova TP Galinski EA Muyzer G Kuenen JG *Thiohalorhabdus denitrificans *gen. nov., sp. nov., an extremely halophilic, sulfur-oxidizing, deep-lineage gammaproteobacterium from hypersaline habitats Int J Syst Evol Microbiol 2008 58 2890 2897 10.1099/ijs.0.2008/000166-0 19060078

[B10] Imhoff JF Suling J The phylogenetic relationship among *Ectothiorhodospiracaea*: a reevaluation of their taxonomy on the basis of 16S rDNA analysies Arch Microbiol 1996 165 106 113 10.1007/s002030050304 8593098

[B11] Imhoff JF Trüper HG *Ectothiorhodospira halochloris *sp. nov., a new extremely halophilic phototrophic bacterium containing bacteriochlorophyll b Arch Microbiol 1977 114 114 121 10.1007/BF00410772

[B12] Lee J Jeon CO Lim J Lee S Lee J Song S Park D Li W Kim C *Halomonas taeanensis *sp. nov., a novel moderately halophilic bacterium isolated from a solar saltern in Korea Int J Syst Evol Microbiol 2005 55 2027 2032 10.1099/ijs.0.63616-0 16166705

[B13] Bowers KJ Mesbah NM Wiegel J *Natranaerobius 'grantii' and Natranaerobius 'jonesii'*, spp. nov., two anaerobic halophilic alkaliphiles isolated from the Kenyan-Tanzanian Rift [abstract] Abst Gen Meet Am Soc Microbiol Boston, MA 2008 I-007

[B14] Sorokin DY Tourova TP Lysenko AM Muyzer G Diversity of culturable halophilic sulfur-oxidizing bacteria in hypersaline habitats Microbiology UK 2006 152 3013 3023 10.1099/mic.0.29106-0 17005982

[B15] Adkins JP Madigan MT Mandelco L Woese CR Tanner RS *Arhodomonas aquaeolei *gen. nov., sp. nov., an aerobic halophilic bacterium isolated from a subterranean brine Int J Syst Evol Microbiol 1993 43 514 520 10.1099/00207713-43-3-514 8347510

[B16] Liaw HJ Mah RA Isolation and characterization of *Haloanaerobacter chitinovorans *gen. nov., sp. nov., a halophilic, anaerobic, chitinolytic bacterium from a solar saltern Appl Env Micro 1992 58 260 266 10.1128/aem.58.1.260-266.1992 PMC195201 16348626

[B17] Wagner ID Wiegel J Wiegel J, Maier RJ, Adams MWW Diversity of Thermophilic Anaerobes Incredible Anaerobes: from Physiology to Genomics to Fuels 2008 Boston: Blackwell Pub. on behalf of the New York Academy of Sciences 1 43 10.1196/annals.1419.029 18378585

[B18] Cayol JL Ollivier B Patel BKC Ageron E Grimont PAD Prensier G Garcia JL *Halanaerobium lacusroseus *sp. nov., an extremely halophilic fermentative bacterium from the sediments of a hypersaline lake Int J Syst Evol Microbiol 1995 45 790 797 10.1099/00207713-45-4-790 7547301

[B19] Padan E Krulwich TA Storz G, Hengge-Aronis R Sodium stress Bacterial Stress Response 2000 Washington, DC.: ASM Press 117 130

[B20] Oren A Halophilic Microorganisms and Their Environments 2002 Dordrecht, the Netherlands: Kluwer Academic Publishers

[B21] Mesbah NM Abou-El-Ela Soad H Wiegel J Novel and unexpected prokaryotic diversity in water and sediments of the alkaline, hypersaline lakes of the Wadi An Natrun, Egypt Microb Ecol 2007 54 598 617 10.1007/s00248-006-9193-y 17450395

[B22] Ghozlan H Deif H Kandil RA Sabry S Biodiversity of moderately halophilic bacteria in hypersaline habitats J Gen Appl Microbiol 2006 52 63 72 10.2323/jgam.52.63 16778349

[B23] Duckworth AW Grant WD Jones BE van Steenbergen R Phylogenetic diversity of soda lake alkaliphiles FEMS 2006 19 181 191

[B24] Mesbah NM Wiegel J *Natronovirga wadinatrunensis *gen. nov., sp. nov. and *Natranaerobius trueperi *sp. nov., two halophilic, alkalithermophilic microorganisms from soda lakes of the Wadi An Natrun, Egypt Int J Syst Evol Microbiol 2009 59 2042 2048 10.1099/ijs.0.008151-0 19605718

[B25] Wiegel J Anaerobic alkalithermophiles, a novel group of extremophiles Extremophiles 1998 2 257 267 10.1007/s007920050068 9783173

[B26] Imhoff JF Trüper HG *Ectothiorhodospira abdelmalekii *sp. nov., a new halophilic and alkaliphilic phototrophic bacterium Zentralbl BakteriolParasitenkd Infektionskr Hyg 1 Orig 1981 C2 228 234

[B27] Zhilina TN Zavarzin GA Detkova EN Rainey FA *Natroniella acetigena *gen. nov. sp. nov., an extremely haloalkaliphilic, homoacetic bacterium: a new member of *Haloanaerobiales* Curr Microbiol 1996 32 320 326 10.1007/s002849900057 8661677

[B28] Antón J Oren A Benlloch S Rodríguez-Valera F Amann R Roselló-Mora R *Salinibacter ruber *gen. nov., sp. nov., a novel, extremely halophilic member of the *Bacteria *from saltern crystallizer ponds Int J Syst Evol Microbiol 2002 52 485 491 11931160 10.1099/00207713-52-2-485

[B29] Vossenberg JLCM Driessen AJM Grant WD Konnings WN Lipid membranes from halophilic and alkali-halophilic *Archaea *have a low H^+ ^and Na^+ ^permeability at high salt concentration Extremophiles 1999 3 253 257 10.1007/s007920050124 10591015

[B30] Hirsch P Hoffman B *Dichotomicrobium thermohalophilum*, gen. nov., spec. nov., budding prosthecate bacteria from the solar lake (Sinai) and some related strains Syst Appl Microbiol 1989 11 291 301

[B31] Raymond JC Sistrom WR *Ectothiorhodospira halophila*, a new species of the genus *Ectothiorhodospira* Archiv fur Mikrobiologie 1969 69 121 126 10.1007/BF00409756 4192367

[B32] Zhilina TN Garnova ES Tourova TP Kostrikina NA Zavarzin GA *Halonatronum saccharophilum *gen. nov., sp. nov.: a new haloalkaliphilic bacterium of the order *Haloanaerobiales *from Lake Magadi Mikrobiologiya 2001 70 77 85 (in Russian). English translation: *Microbiology*, 2001, **70**:64-72 11338841

[B33] Cayol JL Ollivier B Patel BKC Prensier G Guezennec J Garcia JL Isolation and characterization of *Halothermothrix orenii *gen. nov., sp. nov., a halophilic, thermophilic, fermentative, strictly anaerobic bacterium Int J Syst Evol Microbiol 1994 44 534 540 10.1099/00207713-44-3-534 7520742

[B34] Yumoto I Hirota K Sogabe Y Nodasaka Y Yokota Y Hoshino T *Psychrobacter okhotskensis *sp. nov., a lipase-producing facultative psychrophile isolated from the coast of the Okhotsk Sea Int J Syst Evol Microbiol 2003 53 1985 1989 10.1099/ijs.0.02686-0 14657134

[B35] Padan E Venturi M Gerchman Y Dover N Na^+^/H^+ ^antiporters Biochim Biophys Acta 2001 1505 144 157 10.1016/S0005-2728(00)00284-X 11248196

[B36] Roberts MF Organic compatible solutes of halotolerant and halophilic microorganisms Saline Systems 2005 1 5 10.1186/1746-1448-1-5 16176595 PMC1224877

[B37] Mesbah NM Cook GM Wiegel J The halophilic alkalithermophile *Natranaerobius thermophilus *adapts to multiple environmental extremes using a large repertoire of Na^+^(K^+^)/H^+ ^antiporters Mol Microbiol 2009 74 270 281 10.1111/j.1365-2958.2009.06845.x 19708921 PMC2764116

[B38] Foti MJ Sorokin DY Zacharova EE Pimenov NV Kuenen JG Muyzer G Bacterial diversity and activity along a salinity gradient in soda lakes of the Kulunda Steppe (Altai, Russia) Extremophiles 2008 12 133 145 10.1007/s00792-007-0117-7 17989917

[B39] Rees HC Grant WD Jones BE Heaphy S Diversity of Kenyan soda lake alkaliphiles assessed by molecular methods Extremophiles 2004 8 63 71 10.1007/s00792-003-0361-4 15064991

