# Supplementary material for: Biodiversity of poly-extremophilic Bacteria: Does combining the extremes of high salt, alkaline pH and elevated temperature approach a physico-chemical boundary for life?
Source: Saline Syst. 2009 Nov 23;5:9. doi: 10.1186/1746-1448-5-9 (PMC2785825; doi:10.1186/1746-1448-5-9)
Supplement: Additional file 1 — [Na+], pH and Temperature Optima and Ranges for Bacterial Extreme Halophiles. The data provided show the [Na+], pH and temperature optima and ranges for validly published bacterial extreme halophiles. [file 1746-1448-5-9-S1.DOC]

**Additional File 1: [Na^+^], pH and Temperature Optima and Ranges for Bacterial Extreme Halophiles**

| **Genus** | **Species** | **Na+ opt. (M)** | **Na+ range (M)** | **pH opt.** | **pH range** | **Temp. opt.** | **Temp. range** | **Ref.** | **Isolated from** |
| --- | --- | --- | --- | --- | --- | --- | --- | --- | --- |
| **Aerobic** |  |  |  |  |  |  |  |  |  |
|  |  |  |  |  |  |  |  |  |  |
| *Actinopolyspora* | *halophila* | 3.42 | 1.71-5.13 |  |  | 37 | 10-43 | [1] | dairy salt |
| *Alkalibacillus* | *halophilus* | 2.55 | 0.85-5.10 | 7.5 | 6.0-9.0 | 37 | 25-45 | [2] | hypersaline soil Xin-Jiang, China |
| *Arhodomonas* | *aquaeolei* | 2.57 | 1.03-3.42 | 7.0 | 6.0-8.0 | 37 | 20-45 | [3] | petroleum reservoir production fluid |
| *Bacillus* | *persepolensis* | 1.71 | 0.85-3.42 | 8.3 | 7.0-10.0 | 40 | 25-45 | [4] | Howz-Soltan lake, Iran |
| *Dichotomicrobium* | *thermohalophilum* | 2.43 | 1.37-3.80 | 8.5 | 5.8-9.5 | 50 | 20-65 | [5] | solar lake near Elat (Sinai) |
| *Filobacillus* | *milosensis* | 2.39 | 0.34-3.94 | 7.8 | 6.5-8.9 | 38 | -42 | [6] | Palaeochori Bay, Milos, Greece |
| *Gracilibacillus* | *saliphilus* | 2.13 | 0.17-3.74 | 7.0 | 6.0-8.0 | 33 | 4-45 | [7] | Ebinur Lake, China |
| *Haloglycomyces* | *albus* | 1.71 | 0.51-3.06 | 7.25 | 5.0-9.0 | 37 | 15-40 | [8] | Xinjiang Province, China |
| *Halomonas* | *aquamarina* | 1.71 | 0-3.42 | 9.0 | 7.5-10 | 37 | 5-50 | [9,10] | Pacific Ocean |
| *Halomonas* | *campaniensis* | 1.71 | 0-2.7 | 9.0 | 7-10 | 37 | 10-43 | [11] | Malvizza, Campania, Italy |
| *Halomonas* | *campisalis* | 1.5 | 0.2-4.5 | 9.5 | 6.0-11.0 | 30 | 4-50 | [12] | Alkali Lake, Washington, USA |
| *Halomonas* | *cupida* | 1.5 | 0.2-4.5 | 9.5 | 6.0-12.0 | 30 | 4-50 | [13] | marine habitats |
| *Halomonas* | *denitrificans* | 1.71 | 0.34-3.42 | 8.0 | 5.0-10.0 | 35 | 5-50 | [14] | seawater, Anmyeondo, Korea |
| *Halomonas* | *gomseomensis* | 2.05 | 0.17-3.42 | 8.0 | 6.0-10.0 | 30 | 5-45 | [14] | solar saltern, Anmyeondo, Korea |
| *Halomonas* | *gudaonensis* | 2.57 | 0.17-3.42 | 8.0 | 8.0-9.0 | 30 | 10-42 | [15] | oil contaminated saline soil, China |
| *Halomonas* | *ilicicola* | 1.71 | 0.34-2.98 | 6.5 | 6.0-9.0 | 37 | 25-42 | [16] | Santa Pola, Spain |
| *Halomonas* | *janggokensis* | 2.57 | 0.17-3.42 | 8.0 | 6.0-10.0 | 30 | 5-45 | [14] | Janggok saltern, Anmyeondo, Korea |
| *Halomonas* | *maura* | 2.57 | 0.17-3.42 | 7.5 | 6.0-10.0 | 30 | 5-45 | [17] | solar saltern, Asilah, Morocco |
| *Halomonas* | *salaria* | 3.42 | 0-4.28 | 8.0 | 5.0-10.0 | 30 | 10-45 | [14] | seawater, Anmyeondo, Korea |
| *Halomonas* | *shengliensis* | 3.42 | 0-4.28 | 7.5 | 5.0-10.0 | 30 | 10-45 | [18] | Shengli oilfield, China |
| *Halomonas* | *taeanensis* | 2.57 | 0-4.28 | 8.0 | 7.0-10.0 | 35 | 10-45 | [19] | solar saltern, Taean, Korea |
| *Halomonas* | *variabilis* | 1.6 | 1.2-4.9 | 7.5 | 6.5-8.4 | 33 | 15-37 | [20,21] | Great Salt Lake, Utah |
| *Kushneria* | *indalinina* | 1.71 | 0.51-4.28 | 7.2 | 5.0-9.0 | 35 | 10-42 | [22] | solar saltern, Almaria, Spain |
| *Nocardiopsis* | *kunsanenesis* | 1.71 | 0.51-3.42 | 9.0 |  | 37 |  | [23] | saltern, Kunsan, Korea |
| *Nocardiopsis* | *lucentensis* | 1.71 | 0.51-4.28 | 9.0 | 5.0- | 37 |  | [24] | salt marsh, Alicante, Spain |
| *Oceanobacillus* | *kapialis* | 1.71 | 0.09-4.08 | 8.0 | 6.0-9.0 | 37 | 8-43 | [25] | fermented shrimp paste, Thailand |
| *Rhodospirillum* | *sodomense* | 2.05 | 1.03-3.42 | 7.0 |  | 40 | 25-47 | [26] | Dead Sea |
| *Saccharopolyspora* | *halophila* | 2.13 | 0.51-3.42 | 7.5 | 6.0-8.5 | 33 | 10-45 | [27] | Xinjiang Province, China |
| *Saccharopolyspora* | *qijiaojingensis* | 2.13 | 1.02-3.74 | 7.0 | 5.0-8.0 | 28 | 20-40 | [28] | Xinjiang Province, China |
| *Salinibacter* | *ruber* | 4.00 | 2.57- | 8.0 | 6.0-8.5 | 47 | -52 | [29] | saltern crystallizer ponds, Spain |
| *Salinicoccus* | *albus* | 1.71 | 0.17-5.10 | 8.5 | 6.0-10.0 | 25 | 5-40 | [30] | Yunnan, China |
| *Salinicoccus* | *alkaliphilus* | 1.71 | 0-4.28 | 9.0 | 6.5-11.5 | 32 | 10-46 | [31] | Baer Soda Lake, China |
| *Salinicoccus* | *iranensis* | 1.71 | 0.17-4.28 | 7.5 | 6.5-10 | 35 | 5-45 | [32] | textile wastewater, Qom, Iran |
| *Salinicoccus* | *luteus* | 1.71 | 0.17-4.28 | 9.0 | 7.0-11.0 | 30 | 4-45 | [33] | desert soil sample, Egypt |
| *Salinicoccus* | *roseus* | 1.71 | 0.15-4.28 | 8.0 | 6.0-9.0 |  |  | [34] |  |
| *Salinicoccus* | *siamensis* | 1.71 | 0.26-4.28 | 8.5 | 6.0-9.0 | 37 | 15-45 | [35] | fermented shrimp paste |
| **Genus** | **Species** | **Na+ opt. (M)** | **Na+ range (M)** | **pH opt.** | **pH range** | **Temp. opt.** | **Temp. range** | **Ref.** | **Isolated from** |
| **Anaerobic** |  |  |  |  |  |  |  |  |  |
|  |  |  |  |  |  |  |  |  |  |
| *Actinopolyspora* | *iraqiensis* | 2.56 | 0.86-4.27 | 7.5 |  | 35 | 16-40 | [36] | saline soil, Iraq |
| *Halanaerobacter* | *chitinivorans* | 3.0 | 0.5-5.0 | 7.0 |  | 45 | 23-50 | [37] | Solar saltern, California |
| *Halanaerobacter* | *salinarius* | 2.57 | 0.86-5.13 | 7.8 | 5.5-8.5 | 45 | 10-50 | [38] | Salin-de-Giraud, France |
|  |  |  |  |  |  |  |  |  |  |
| *Halanaerobaculum* | *tuisiense* | 3.57 | 2.38-5.10 | 7.3 | 5.9-8.4 | 42 | 30-50 | [39] | El-Djerid Chott, Tunisia |
| *Halanaerobium* | *kushneri* | 2.05 | 1.54-3.08 | 7.5 | 6.0-8.0 | 40 | 20-45 | [40] | reservoir production fluid, Oklahoma |
| *Halanaerobium* | *lacusrosei* | 3.42 | 1.03-5.82 | 7.0 |  | 40 | 20-50 | [18] | Retba Lake, Senegal |
| *Halanaerobium* | *praevalens* | 2.14 | 0.34-5.13 | 7.2 | 6.0-9.0 | 37 | 5-50 | [42] | Great Salt Lake, Utah |
| *Halanaerobium* | *saccharolyticum* | 1.7 | 0.51-5.1 | 7.5 | 6.0-8.0 | 40 | 15-47 | [43,44] | Lake Civash |
|  | *subsp. saccharolyticum* |  |  |  |  |  |  |  |  |
| *Halanaerobium* | *saccharolyticum* | 2.1 | 0.8-4.3 | 7.0 | 6.3-8.7 | 40 | 20-47 | [43,44] | Retba Lake, Senegal |
|  | *subsp. senegalense* |  |  |  |  |  |  |  |  |
| *Halocella* | *cellulosilytica* | 2.57 | 0.86-3.42 | 7.0 | 5.5-8.5 | 39 | 20-50 | [45] | Lake Sivash, France |
| *Halonatronum* | *saccharophilum* | 2.05 | 0.51-2.91 | 8.5 | 7.7-10.3 | 55 | 18-60 | [46] | Lake Magadi, Kenya |
| *Halorhodospira* | *abdelmalekii* | 3.08 | 0.86-5.13 | 9.2 |  | 44 |  | [47,48] |  |
| *Halorhodospira* | *halochloris* | 4.62 | 1.71-5.81 | 8.5 | 8.1-9.1 | 48 | 33-50 | [49,48] | Wadi An Natrun, Egypt |
| *Halorhodospira* | *halophila* | 3.76 | 1.54-5.14 | 7.8 |  | 50 |  | [50,48] |  |
| *Halothermothrix* | *orenii* | 1.71 | 0.68-3.42 | 7.0 | 5.5-8.2 | 60 | 45-68 | [51] | Chott El Guettar (lake), Tunisia |
| *Natranaerobius* | *‘grantii’* | 4.3 | 2.9-sat. | 9.5 | 7.5-10 | 46 | 31-52 | [52] | Lake Magdi, Kenya |
| *Natranaerobius* | *‘jonesii’* | 3.9 | 3.1-5.0 | 10.5 | 8.5-11.5 | 66 | 47-71 | [52] | Lake Magdi, Kenya |
| *Natranaerobius* | *thermophilus* | 3.9 | 3.1-4.9 | 9.5 | 8.3-10.6 | 53 | 35-56 | [53] | Wadi An Natrun, Egypt |
| *Natranaerobius* | *trueperi* | 3.7 | 3.1-5.4 | 9.5 | 7.8-11.0 | 52 | 26-55 | [54] | Wadi An Natrun, Egypt |
| *Natroniella* | *acetigena* | 2.57 | 1.71-3.42 | 9.7 | 8.1-10.7 | 37 | -42 | [55] | Lake Magdi, Kenya |
| *Natronovirga* | *wadinatrunensis* | 3.9 | 3.1-5.3 | 9.9 | 8.5-11.5 | 51 | 24-58 | [54] | Wadi An Natrun, Egypt |
|  |  |  |  |  |  |  |  |  |  |
| **Facultatively anaerobic** | |  |  |  |  |  |  |  |  |
|  |  |  |  |  |  |  |  |  |  |
| *Halomonas* | *sinaiensis* | 2.57 | 0.86-5.13 | 7.0 | 6.0-9.0 | 35 | 25-50 | [56] | Ras Muhammad Park, Egypt |
| *Halospina* | *denitrificans* | 3.0 | 2.0-5.0 |  | 6.7-8.5 |  |  | [21] |  |
| *Halovibrio* | *denitrificans* | 2.25 | 2.0-5 |  | 6.7-8.5 |  |  | [21] | hypersaline lake, Mongolia |
| *Salinivibrio* | *siamensis* | 1.71 | 0.17-3.74 | 8.0 | 5.0-9.0 | 37 | 10-47 | [57] | fermented fish, Thailand |
| *Thiohalorhabdus* | *denitrificans* | 3.0 | 2.0-5.0 | 7.8 | 6.5-7.2 | 35 |  | [58] | hypersaline lake, Siberia, Russia |

^a^blank cells indicate data not found

^b^Shaded cells indicate data meeting or exceeding one of the following criteria:

[Na^+^]_opt_ ≥ 2.0 M, pH_opt_ ≥ 8.5, T_opt_ ≥ 50˚C

1. Gochnauer MB, Leppard GG, Komaratat P, Kates M, Novitsky T, Kushner DJ: **Isolation and characterization of *Actinopolyspora* *halophila*, gen. et sp. nov., an extremely halophilic actinomycete**. *Can* *J* *Microbiol* 1975, **21:**1500-1511.
2. Tiang XP, Dastager SG, Lee JC, Tang SK, Zhang YQ, Park DJ, Kim CJ, Li WJ: ***Alkalibacillus halophilus* sp. nov., a new halophilic species isolated from hypersaline soil in Xin-Jiang province, China.** *Syst Appl Microbiol* 2007, **30**:268-272.
3. Adkins JP, Madigan MT, Mandelco L, Woese CR, Tanner RS: ***Arhodomonas aquaeolei* gen. nov., sp. nov., an aerobic halophilic bacterium isolated from a subterranean brine.** *Int J Syst Ev Microbiol* 1993, **43:**514-520.
4. Amoozegar MA, Sánchez-Porro C, Rohban R, Hajighasemi M, Ventosa A: ***Bacillus persepolensis* sp. nov., a moderately halophilic bacterium from a hypersaline lake.** *Int J Syst Evol Microbiol* 2009, **59**:2352-2358.
5. Hirsch P, Hoffman B: ***Dichotomicrobium thermohalophilum*, gen. nov., spec. nov., budding prosthecate bacteria from the solar lake (Sinai) and some related strains.** *Syst Appl Microbiol* 1989, **11:**291-301.
6. Schlesner H, Lawson PA, Collins MD, Weiss N, Wehmeyer U, Völker H, Thomm M: ***Filobacillus* *milensis* gen. nov., sp. nov., a new halophilic spore-forming bacterium with Orn-D-Glu-type peptidoglycan**. *Int* *J* *Syst* *Evol* *Microbiol* 2001, **51:**425-431.
7. Tang SK, Wang Y, Lou K, Mao PH, Jin X, Jiang CL, Xu LH, Li WJ: ***Gracibacillus saliphilus* sp. nov., a moderately halophilic bacterium isolated from a salt lake.** *Int J Syst Evol Microbiol* 2009, **59**:1620-1624.
8. Guan TW, Tang SK, Wu JY, Zhi XY, Xu LH, Zhang LL, Li WJ: ***Haloglycomyces albus* gen. nov., sp. nov., a halophilic, filamentous actinomycete of the family *Glycomycetaceae*.** *Int J Syst Evol Microbiol* 2009, **59**:1297-1301.
9. Akagawa M, Yamasoto K: **Synonymy of *Alcaligenes* *aquamarinus*, *Alcaligenes* *faecalis* subsp. *homari*, and *Deleya* *aesta*: *Deleya* *aquamarina* comb. nov. as the type species of the genus *Deleya*.** *Int* *J* *Syst* *Bacteriol* 1989, **39:**462-466.
10. Dobson SJ, Franzmann PD: **Unification of the genera *Deleya* (Baumann *et* *al*. 1983), *Halomonas* (Vreeland *et* *al*. 1980), and *Halovibrio* (Fendrich 1988) and the species *Paracoccus* *halodenitrificans* (Robinson and Gibbons 1952) into a single genus, *Halomonas*, and placement of the genus *Zymobacter* in the family *Halomonadaceae*.** *Int* *J* *Syst* *Bacteriol* 1996, **46**:550-558.
11. Romano I, Giordano A, Lama L, Nicolaus B, Gambacorta A: ***Halomonas campaniensis* sp. nov., a haloalkaliphilic bacterium isolated from a mineral pool of Campania Region, Italy.** *Syst Appl Microbiol* 2005, **28:**610-618.
12. Mormile MR, Romine MF, Garcia MT, Ventosa A, Bailey TJ, Peyton BM: ***Halomonas* *campisalis* sp. nov., a denitrifying, moderately haloalkaliphilic bacterium.** *Syst* *Appl*  *Microbiol* 1999, **22:**551-558.
13. Baumann L, Bowditch RD, Baumann P: **Description of *Deleya* gen. nov. created to accommodate the marine species *Alcaligenes* *aestus*, *A*. *pacificus*, *A*. *cupidus*, *A*. *venustus*, and *Pseudomonas* *marina*.** *Int* *J* *Syst* *Bacteriol* 1983, **33**:793-802.
14. Kim KK, Jin L, Yang HC, Lee S**: *Halomonas gomseomensis* sp. nov., *Halomonas janggokensis* sp. nov., *Halomonas salaria* sp. nov.and *Halomonas denitrificans* sp. nov., moderately halophilic bacteria isolated from saline water.** *Int J Syst Ev Microbiol 2007,* ***57:***675-681*.*
15. Wang YN, Cai H, Yu SL, Wang ZY, Liu J, Wu XL: ***Halomonas* *gudaonensis* sp. nov., isolated from a saline soil contaminated by crude oil.** *Int* *J* *Syst* *Evol* *Microbiol*, 2007, **57:**911-915.
16. Arenas M, Banon PI, Copa-Patino JL, Sánchez-Porro C, Ventosa A, Soliveri J: ***Halomonas ilicicola* sp. nov., a moderately halophilic bacterium isolated from a saltern.** *Int J Syst Evol Microbiol* 2009, **59**:578-582.
17. Bouchotroch S, Quesada E, del Moral A, Llamas I, Béjar V: ***Halomonas* *maura* sp. nov., a novel moderately halophilic, exopolysaccharide-producing bacterium.** *Int* *J* *Syst* *Evol* *Microbiol* 2001, **51:**1625-1632.
18. Wang YN, Cai H, Chi CQ, Lu AH, Lin XG, Jiang ZF and Wu XL: ***Halomonas* *shengliensis* sp. nov., a moderately halophilic, denitrifying, crude-oil-utilizing bacterium.** *Int* *J* *Syst* *Evol* *Microbiol* 2007, **57**:1222-1226.
19. Lee J, Jeon CO, Lim J, Lee S, Lee J, Song S, Park D, Li W, Kim C: ***Halomonas taeanensis* sp. nov., a novel moderately halophilic bacterium isolated from a solar saltern in Korea.** *Int J Syst Ev Microbiol* 2005, **55:**2027-2032.
20. Fendrich C**: *Halovibrio variabilis* gen. nov. sp. nov., *Pseudomonas halophila* sp. nov. and a new halophilic aerobic coccoid *Eubacterium* from Great Salt Lake, Utah, USA**. *Syst. Appl. Microbiol*. 1988, **11:**36-43.
21. Sorokin DY, Tourova TP, Galinski EA, Belloch C, Tindall BJ: **Extremely halophilic denitrifying bacteria from hypersaline inland lakes, *Halovibrio* *denitrificans* sp. nov. and *Halospina* *denitrificans* gen. nov., sp. nov., and evidence that the genus name *Halovibrio* Fendrich 1989 with the type species *Halovibrio* *variabilis* should be associated with DSM 3050.** *Int* *J* *Syst* *Evol* *Microbiol* 2006, **56**:379-388.
22. Cabrera A, Aguilera M, Fuentes S, Incerti C, Russell NJ, Ramos-Cormenzana A, Monteoliva-Sánchez M: ***Halomonas* *indalinina* sp. nov., a moderately halophilic bacterium isolated from a solar saltern in Cabo de Gata, Almeria, southern Spain**. *Int* *J* *Syst* *Evol* *Microbiol* 2007, **57:**376-380.
23. Chun J, Bae KS, Moon EY, Jung SO, Lee HK, Kim SJ: ***Nocardiopsis* *kunsanensis* sp. nov., a moderately halophilic actinomycete isolated from a saltern.** *Int* *J* *Syst* *Evol* *Microbiol* 2000, **50**:1909-1913.
24. Yassin AF, Galinski EA, Wohlfarth A, Jahnke KD, Schaal KP, Trür HG: **A new actinomycete species, *Nocardiopsis* *lucentensis* sp. nov***. Int* *J* *Syst* *Evol* *Microbiol*  1993, **43**:266-271.
25. Namwong S, Tanasupawat S, Lee KC, Lee JS: ***Oceanobacillus kapialis* sp. nov., from fermented shrimp paste in Thailand.** *Int J Syst Evol Microbiol* 2009, **59**:2254-2259.
26. Mack EE, Mandelco L, Woese CR, Madigan MT: ***Rhodospirillum* *sodomense*, sp. nov., a Dead Sea *Rhodospirillum* species.** *Arch* *Microbiol* 1993, **160**:363-371.
27. Tang SK, Wang Y, Cai M, Zhi XY, Lou K, Xu LH, Jiang CL, Li WJ: ***Saccharopolyspora halophila* sp. nov., a novel halophilic actinomycete isolated form a saline lake in China.** *Int J Syst Evol Microbiol* 2009, **59**:555-558.
28. Tang SK, Wang Y, Wu JY, Cao LL, Lou K, Xu LH, Jiang CL, Li WJ: ***Saccharopolyspora qijiaojingensis* sp. nov., a halophilic actinomycete isolated from a salt lake.** *Int J Syst Evol Microbiol 2009,* ***59:****2166-2170.*
29. Antón J, Oren A, Benlloch S, Rodríguez-Valera F, Amann R, Roselló-Mora R: ***Salinibacter ruber* gen. nov., sp. nov., a novel, extremely halophilic member of the *Bacteria* from saltern crystallizer ponds.** *Int J Syst Ev Microbiol* 2002, **52:**485-491.
30. Chen YG, Cui XL, Wang YX, Zhang YQ, Li QY, Liu ZX, Wen ML, Peng Q, Li WJ: ***Salinicoccus albus* sp. nov., a halophilic bacterium from a salt mine.** *Int J Syst Evol Microbiol* 2009, **59:**874-879.
31. Zhang W, Xue Y, Ma Y, Zhou P, Ventosa A, Grant WD: ***Salinicoccus* *alkaliphilus* sp. nov., a novel alkaliphile and moderate halophile from Baer Soda Lake in Inner Mongolia Autonomous Region, China.** *Int* *J* *Syst* *Evol* *Microbiol* 2002, **52**:789-793.
32. Amoozegar MA, Schumann P, Hajighasemi M, Ashengroph M, Razavi MR: ***Salinicoccus* *iranensis* sp. nov., a novel moderate halophile.** *Int* *J* *Syst* *Evol* *Microbiol* 2008, **58**:178-183.
33. Zhang YQ, Yu LY, Liu HY, Zhang YQ, Xu LH, Li WJ: ***Salinicoccus* *luteus* sp. nov., isolated from a desert soil.** *Int* *J* *Syst* *Evol* *Microbiol* 2007, **57**:1901-1905.
34. Ventosa A, Marquez MC, Ruiz-Berraquero F, Kocur M: ***Salinicoccus* *roseus* gen. nov., sp. nov., a new moderately halophilic gram-positive coccus.** *Syst* *Appl* *Bacteriol* 1990, **13**:29-33.
35. Pakdeeto A, Tanasupawat S, Thawai C, Moonmangmee S, Kudo T, Itoh T: ***Salinicoccus siamensis* sp. nov., isolated from fermented shrimp paste in Thailand.** *Int J Syst Ev Microbiol* 2007, **57:**2004-2008.
36. Ruan JS, Al-Tai AM, Zhou ZH, Qu LH: ***Actinopolyspora* *iraqiensis* sp. nov., a new halophilic actinomycete isolated from soil.** *Int* *J* *Syst* *Evol* *Microbiol* 1994, **44**:759-763.
37. Liaw HJ, Mah RA: **Isolation and characterization of *Haloanaerobacter chitinovorans* gen. nov., sp. nov., a halophilic, anaerobic, chitinolytic bacterium from a solar saltern.** *Appl Env Micro* 1992, **58:**260-266.
38. Mouné S, Manac’h N, Hirschler A, Caumette P, Willison JC, Matheron R: ***Haloanaerobacter salinarius* sp. nov., a novel halophilic fermentative bacterium that reduces glycine-betaine to trimethylamine with hydrogen or serine as electron donors; emendation of the genus *Haloanaerobacter.*** *Int J Syst Ev Microbiol 1999,* ***49****:103-112.*
39. Hedi A, Fardeau ML, Sadfi N, Boudabous A, Ollivier B, Cayol JL: **Characterization of *Halanaerobaculum tunisiense* gen. nov., sp. nov., a new halophilic fermentative, strictly anaerobic bacterium isolated from a hypersaline lake in Tunisia.** *Extremophiles* 2009, **13**:313-319.
40. Bhupathiraju VK, McInerney MJ, Woese CR,Tanner RS: ***Haloanaerobium* *kushneri* sp. nov., an obligately halophilic, anaerobic bacterium from an oil brine.** *Int* *J* *Syst* *Evol* *Microbiol* 1999, **49**:953-960.
41. Cayol JL, Ollivier B, Patel BKC, Ageron E, Grimont PAD, Prensier G, Garcia JL: ***Halanaerobium lacusroseus* sp. nov., an extremely halophilic fermentative bacterium from the sediments of a hypersaline lake.** *Int J Syst Evol Microbiol* 1995, **45:**790-797.
42. Zeikus G, Hegge PW, Thompson TE, Phelps TJ, Langworthy TA: **Isolation and description of *Haloanaerobium* *praevalens* gen. nov. and sp. nov., an obligately anaerobic halophile common to Great Salt Lake sediments.** *Curr* *Microbiol* 1983, **9**:225-234.
43. Cayol JL, Ollivier B, Soh ALA, Fardeau ML, Ageron E, Grimont PAD, Prensier G, Guezennec J, Magot M, Garcia JL: ***Haloincola* *saccharolytica* subsp. *senegalensis* subsp. nov., isolated from the sediments of a hypersaline lake, and emended description of *Haloincola* *saccharolytica*.** *Int* *J* *Syst* *Evol* *Microbiol* 1994, **44**:805-811.
44. ZhilinaTN, Zavarzin GA, Bulygina ES, Kevbrin VV, Osipov GA, Chumakov KM: **Ecology, physiology and taxonomy studies on a new taxon of *Haloanaerobiaceae*, *Haloincola* *saccharolytica* gen. nov., sp. nov.** *Syst* *Appl*. *Microbiol* 1992, **15**:275-284.
45. Simankova MV, Chernych NA, Osipov GA, Zavarzin GA: ***Halocella* *cellulolytica* gen. nov., sp. nov., a new obligately anaerobic, halophilic, cellulolytic bacterium.** *Syst* *Appl* *Microbiol* 1993, **16**:385-389.
46. Zhilina TN, Garnova ES, Tourova TP, Kostrikina NA, Zavarzin GA: ***Halonatronum* *saccharophilum* gen. nov., sp. nov.: a new haloalkaliphilic bacterium of the order *Haloanaerobiales* from Lake Magadi.** *Mikrobiologiya* 2001, **70**:77-85 (in Russian). English translation: *Microbiology*, 2001, **70**:64-72.
47. Imhoff JF, Trüper HG: ***Ectothiorhodospira* *abdelmalekii* sp. nov., a new halophilic and alkaliphilic phototrophic bacterium.** *Zentralbl* *Bakteriol* *Parasitenkd* *Infektionskr* *Hyg* *Abt* *1* *Orig* 1981, **C2**:228-234.
48. Imhoff JF, Suling J: **The phylogenetic relationship among *Ectothiorhodospiracaea*: a reevaluation of their taxonomy on the basis of 16S rDNA analysies.** *Arch Microbiol* 1996, **165**:106-113.
49. Imhoff JF, Trüper HG: ***Ectothiorhodospira* *halochloris* sp. nov., a new extremely halophilic phototrophic bacterium containing bacteriochlorophyll b**. *Arch* *Micro* 1977, **114**:114-121.
50. Raymond JC, Sistrom WR: ***Ectothiorhodospira* *halophila*, a new species of the genus *Ectothiorhodospira*.** *Archiv* *fur* *Mikrobiologie* 1969, **69**:121-126.
51. Cayol JL, Ollivier B, Patel BKC, Prensier G, Guezennec J and Garcia JL: **Isolation and characterization of *Halothermothrix* *orenii* gen. nov., sp. nov., a halophilic, thermophilic, fermentative, strictly anaerobic bacterium.** *Int* *J* *Syst* *Evol* *Microbiol* 1994, **44**:534-540.
52. Bowers KJ, Mesbah NM, Wiegel J: ***Natranaerobius ‘grantii’ and Natranaerobius ‘jonesii’*, spp. nov., two anaerobic halophilic alkaliphiles isolated from the Kenyan-Tanzanian Rift [abstract].** *ASM General Meeting 2008 Boston, MA,* I-007.
53. Mesbah NM, Hedrick DB, Peacock AD, Rohde M, Wiegel, J: ***Natranaerobius thermophilus* gen. nov., sp. nov., a halophilic, alkalithermophilic bacterium from soda lakes of the Wadi An Natrun, Egypt, and proposal of *Natranaerobiaceae* fam. nov. and *Natranaerobiales* ord. nov.** *Int J Syst Ev Micro* 2007, **57:**2507-2512.
54. Mesbah NM, Wiegel J: ***Natronovirga wadinatrunensis* gen. nov., sp. nov. and *Natranaerobius trueperi* sp. nov., two halophilic, alkalithermophilic microorganisms from soda lakes of the Wadi An Natrun, Egypt.** *Int J Syst Ev Microbiol* 2009, **59:**2042-2048.
55. Zhilina TN, Zavarzin GA, Detkova EN, Rainey FA: ***Natroniella* *acetigena* gen. nov. sp. nov., an extremely haloalkaliphilic, homoacetic bacterium: a new member of *Haloanaerobiales*.** *Curr* *Microbiol* 1996, **32**:320-326.
56. Romano I, Lama L, Orlando P, Nicolaus B, Giordamo A: ***Halomonas sinaiensis* sp. nov., a novel halophilic bacterium isolated from a salt lake inside Ras Muhammad Park, Egypt.** *Extremophiles* 2007, **11:**789-786.
57. Chamroensaksri N, Tanasuawat S, Akaracharanya A, Visessanguan W, Kudo T, Itoh T: ***Salinivibrio siamensis* sp. nov., from fermented fish (*pla-ra*) in Thailand.** *Int J Syst Evol Microbiol* 2009, **59:**880-885.
58. Sorokin DY, Tourova TP, Galinski EA, Muyzer G, Kuenen JG: ***Thiohalorhabdus denitrificans* gen. nov., sp. nov., an extremely halophilic , sulfur-oxidizing, deep-lineage gammaproteobacterium from hypersaline habitats.** *Int J Syst Evol Microbiol* 2008, **58**:2890-2897.
